# Causal Link between Gut Microbiota, Neurophysiological States, and Bone Diseases: A Comprehensive Mendelian Randomization Study

**DOI:** 10.3390/nu15183934

**Published:** 2023-09-11

**Authors:** Shaoting Luo, Zhiyang Chen, Linfang Deng, Yufan Chen, Weizheng Zhou, Federico Canavese, Lianyong Li

**Affiliations:** 1 Department of Pediatric Orthopedics, Shengjing Hospital of China Medical University, Shenyang 110004, China; lst634747560@163.com (S.L.); chenyfchaos1121@163.com (Y.C.); cmuzwz@163.com (W.Z.); 2 Department of Otolaryngology Head and Neck Surgery, Shengjing Hospital of China Medical University, Shenyang 110004, China; chenzhiyang18@gmail.com; 3 Department of Nursing, Jinzhou Medical University, Jinzhou 121001, China; 4 Department of Pediatric Orthopedic Surgery, Lille University Centre, Jeanne de Flandre Hospital, 59000 Lille, France; federico.canavese@univ-lille.fr

**Keywords:** fracture, osteoarthritis, cognitive performance, Mendelian randomization, mediation, gut microbiome

## Abstract

Increasing evidence highlights a robust correlation between the gut microbiota and bone diseases; however, the existence of a causal relationship between them remains unclear. In this study, we thoroughly examined the correlation between gut microbiota and skeletal diseases using genome-wide association studies. Linkage disequilibrium score regression and Mendelian randomization were used to probe genetic causality. Furthermore, the potential mediating role of neuropsychological states (i.e., cognition, depression, and insomnia) between the gut microbiota and bone diseases was evaluated using mediation analysis, with genetic colocalization analysis revealing potential targets. These findings suggest a direct causal relationship between Ruminococcaceae and knee osteoarthritis (OA), which appears to be mediated by cognitive performance and insomnia. Similarly, a causal association was observed between Burkholderiales and lumbar pelvic fractures, mediated by cognitive performance. Colocalization analysis identified a shared causal variant (rs2352974) at the TRAF-interacting protein locus for cognitive ability and knee OA. This study provides compelling evidence that alterations in the gut microbiota can enhance cognitive ability, ameliorate insomnia, and potentially reduce the risk of site-specific fractures and OA. Therefore, strategies targeting gut microbiota optimization could serve as novel and effective preventive measures against fractures and OA.

## 1. Introduction

With advancing age, the human body becomes more susceptible to numerous bone health complications [1], of which osteoarthritis (OA) and fractures are particularly debilitating [2]. These conditions profoundly affect the quality of life of older adults, a concern that cannot be overstated [3]. OA is a chronic degenerative disease that manifests as pain, stiffness, swelling, and ultimately, the loss of normal joint function [4]. Fractures, particularly those occurring in weight-bearing areas, such as the spine, pelvis, and hip, can inflict severe acute pain, cause chronic discomfort, and reduce mobility, causing considerable loss of independence [5]. This loss particularly impacts older adults who may already be adversely affected by other health conditions [6]. Consequently, these prevalent health issues pose a significant burden on global healthcare systems and present formidable challenges to patients, families, and caregivers [7]. Considering the substantial physical and psychological impacts of these conditions, along with significant associated treatment costs and the lack of effective long-term therapies [8,9], exploring innovative preventive strategies and developing more effective interventions is critical.

In the continually evolving realm of human health research, the role of the gut microbiota has emerged as a central focus for extensive investigations [10]. These microbial communities perform essential functions [11], including maintaining digestive homeostasis, regulating immune responses [12], and synthesizing vital nutrients. Multiple studies have established a direct connection between the gut microbiota and bone health [13,14,15]. For example, certain gut microbiota can produce short-chain fatty acids, such as butyrate, propionate, and acetate, which play pivotal roles in bone health [16]. Specifically, butyrate has been found to enhance osteoblast function and inhibit osteoclast activity, thereby promoting overall bone health [17]. Furthermore, the gut microbiota can indirectly influence bone health via immune system regulation by affecting T cell and regulatory T cell ratios, which are crucial factors in the development of osteoporosis [18].

As our understanding of the role of the gut microbiota expands, the intriguing concept of the gut–brain axis has emerged [19]. This complex network of biological communication suggests that alterations in the gut microbial community can significantly affect the neurophysiological state [20]. These alterations can manifest in diverse ways, affecting areas such as emotion regulation, sleep patterns, and cognitive functions [21]. Changes in neurophysiological states, such as cognitive decline, depression, and insomnia, are also associated with an increased risk of bone diseases, including osteoporosis and OA [22,23]. However, the underlying mechanisms are likely multifaceted and involve changes in neuroendocrine and immune responses [24].

Neurophysiological factors, such as cognitive performance, emotional regulation, and sleep patterns, could mediate the influence of gut microbiota on bone health, thereby affecting conditions such as OA and fractures [25]. However, the exact mechanisms underlying these relationships and the potential mediating role of neurophysiology require further clarification. 

To comprehensively examine this hypothesis, we applied a Mendelian randomization (MR) analysis, which uses genetic variations as instrumental variables to infer unobservable causal relationships in observational studies. MR provides a robust defense against confounding factors and bias, thereby offering an accurate portrayal of the gut microbiota–bone health relationship [26,27].

Notably, existing studies have often restricted their scope to isolated factors such as inflammation [28], genetics [29], or lifestyle [30]. This narrow focus has culminated in fragmented understanding, which consequently limits advancements in therapeutic approaches. Our research diverges from this trend by pioneering an investigation into the gut–brain–bone axis. By utilizing the methodological rigor of MR analysis, this study not only promises a holistic understanding of the interconnected mechanisms underlying bone diseases but also unveils previously unexplored avenues for both preventive and therapeutic interventions. This multidisciplinary lens uniquely positions our research at the frontier of scientific innovation, conferring the potential to substantially shift prevailing paradigms.

In summary, the primary objective of this study was to examine the gut–brain–bone axis, which is an emerging research field in contemporary medicine [31]. This complex relationship, which involves the gut microbiota, cognitive performance, depression, insomnia, and bone diseases, may provide a foundation for innovative preventive and therapeutic strategies. By elucidating the intricate interactions between the gut microbiota and bone diseases, this study seeks to enhance our understanding of OA and fractures, thereby allowing the exploration of new avenues for their prevention and management in the aging population.

## 2. Materials and Methods

**Figure 1:**
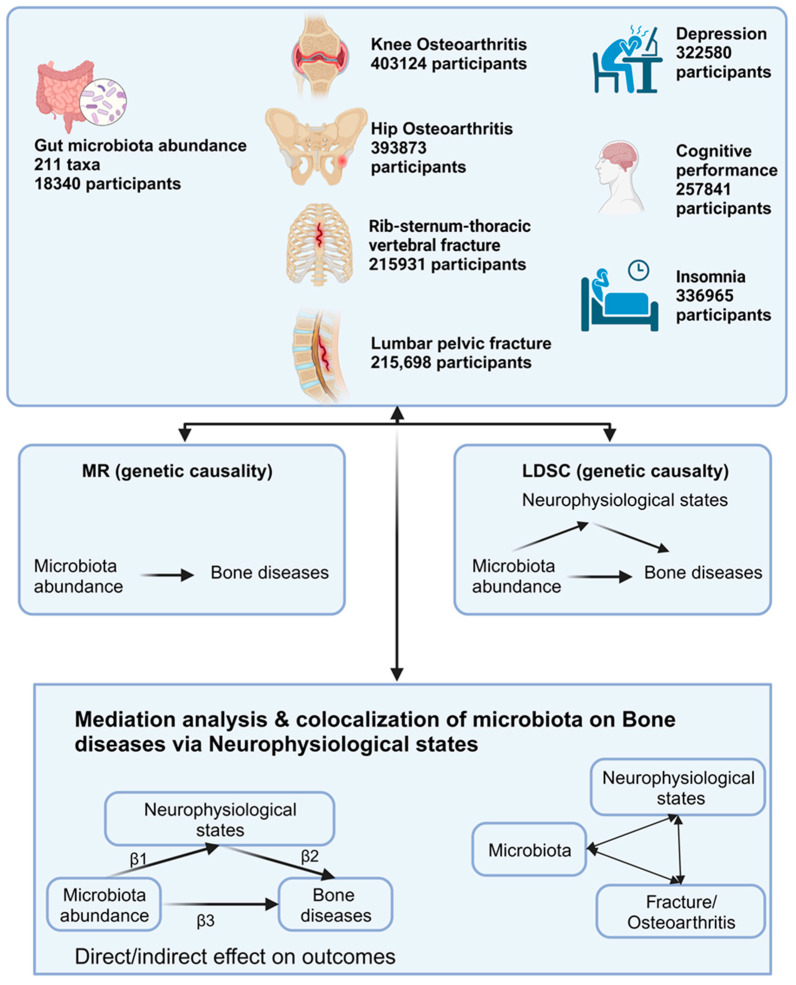
Comprehensive study design and flowchart. Visual representation of the extensive experimental design used in this study, featuring methodologies such as Mendelian randomization (MR), linkage disequilibrium score regression (LDSC), mediation analysis, and gene colocalization.

Summary statistics on the gut microbiota, cognitive performance, depression, insomnia, OA, and fracture risk were obtained from the respective consortia for the study design. Subsequently, large-scale MR and linkage disequilibrium score regression (LDSC) [32] were applied to probe the genetic causality and correlation between gut microbiota abundance and bone diseases, particularly fractures and OA. Finally, mediation analysis [33] and colocalization were employed to examine the interactions between the gut microbiota, neurophysiological states, and bone diseases [34]. STROBE-MR guidelines were followed for the MR analysis, with several methods adopted to uphold the three fundamental assumptions of MR (Figure 1) [35]. The present study was conducted from 7 May to 12 August 2023. Data banks were consulted between 1 July and 1 August 2023.

### 2.1. Instrument Variables Selection

Summary statistics relating to gut microbiota abundance were derived from a genome-wide association study (GWAS) encompassing 18,340 mixed-ancestry participants, 85% of whom were of European descent. These data were sourced from the MiBioGen consortium and included 211 taxa: 9 phyla, 16 classes, 20 orders, 35 families, and 131 genera were identified using 16S ribosomal RNA gene sequencing [36].

A compilation of single-nucleotide polymorphisms (SNPs) related to cognitive functioning was drawn from two principal sources: the UK Biobank and Cognitive Genomics Consortium datasets. These cumulative datasets offer a broad overview, comprising approximately 10 million unique genetic variations discovered in 257,841 individuals of predominantly European ancestry. The involvement of these individuals in GWAS was primarily centered on educational accomplishments. The cognitive assessments selected included digit symbol coding, digit span, word reading, semantic fluency, visual memory, vocabulary, verbal memory of words, verbal memory of stories, phonemic fluency, and a trail-making test. The variety of these assessments afforded a comprehensive understanding of cognitive performance in relation to educational achievement. This understanding laid the groundwork for subsequent analyses using this dataset [37].

Summary statistics for depression were derived from the latest GWAS involving broad depression, which was conducted on UK Biobank participants. This study included a sample size of 322,580 individuals. In this study, broad depression was characterized by past self-reported incidents of seeking help for issues associated with nerves, anxiety, tension, or depression [38].

Furthermore, summary statistics for insomnia were acquired from the latest public GWAS release, which comprised over 10 million genetic variants derived from 109,402 insomnia patients and 277,131 control subjects from the UK Biobank. Insomnia cases were determined based on the response to the question, “Do you have trouble falling asleep at night or do you wake up in the middle of the night?” Participants were provided five potential answers: “I don’t know,” “never/rarely,” “sometimes,” “usually,” and “prefer not to answer.” Participants who responded with “usually” were classified as insomniacs, whereas those responding with “never/rarely” or “sometimes” were categorized as control subjects [39].

To augment the statistical power of genetic loci identification, a comprehensive definition of fractures was implemented. This approach defines fracture cases as those individuals who have experienced fractures at any skeletal location. These cases were verified using a combination of medical records, radiological evidence, and self-reported questionnaire responses. This robust definition and validation process ensured a high probability of identifying significant genetic loci related to fracture incidence. Genetic data related to the location of the fractures used in the GWAS were acquired from the FinnGen Consortium. The lumbar pelvic fracture data comprised 2859 cases and 212,839 controls, whereas the rib–sternum–thoracic vertebral fracture data involved 4070 patients and 211,861 controls. The most comprehensive GWAS summary data pertaining to OA susceptibility were procured from the dataset hosted by the European Bioinformatics Institute. This dataset included substantial sample sizes of 393,873 and 403,124 patients with hip and knee OA, respectively. All participants in this dataset were of European ancestry, thus enabling a large-scale and focused study of the genetic factors influencing OA susceptibility in this population.

The process of selecting instrumental variables (IVs) for exposure in MR adhered to certain guidelines [40]. For every microbiota taxon and pathway, variants that exhibited a genome-wide significance level of *p* < 1 × 10^−5^ and effect allele frequency (EAF) of >0.01 were included [41]. These genetic variants were subjected to a clumping process using a linkage disequilibrium threshold of r^2^ < 0.001 by leveraging the 1000 Genomes European reference panel. F-statistics were computed to mitigate the risk of weak instrument bias. The remaining genetic variants were subsequently used as IVs to model the influence of specific gut microbiota taxa and pathways. In relation to cognitive performance, insomnia, and depression, SNPs exhibiting a genome-wide significance level of *p* < 5 × 10^−8^ and EAF exceeding 0.01 were included. All SNPs were subjected to a clumping process with an LD threshold of r^2^ < 0.001 using the 1000 Genomes European reference panel.

### 2.2. Genetic Causality and Correlation of Microbiota and Bone Diseases

MR was used to quantify the causal effect by employing the inverse-variance weighted (IVW) method. The estimation was expressed as an effect size (β) along with a 95% confidence interval (CI). Cochran’s Q test was used to assess the heterogeneity of effects. Various sensitivity analyses were performed using various methods, such as weighted-median, mode-based, MRPRESSO, and contamination mixture, to verify the outcomes derived from the IVW method. The MR-Egger intercept was used to examine the potential horizontal pleiotropy. Any results exhibiting a *p* value < 0.05 for the Egger intercept were excluded from the study.

Additionally, LDSC was used to determine the heritability of 211 microbial taxa and mediators associated with OA and fractures. LD scores were determined for all high-quality genetic variants (i.e., those with an INFO score > 0.9 and EAF > 0.01) originating from each GWAS. The heritability of specific traits, excluding certain gut microbiota, was calculated.

To expand our understanding of the genetic correlation, LDSC was conducted separately on the 211 microbiota taxa and the 3 mediators (insomnia, depression, and cognitive performance) in relation to both OA and fractures based on GWAS summary statistics. The genetic correlation between OA and fractures was also calculated to estimate genetic similarity within the two independent cohorts.

### 2.3. Mediation Analysis and Colocalization

Two-step MR was conducted to investigate whether neurophysiological states play a mediating role between the gut microbiota, OA, and fractures. The initial step entailed estimating the causal effect of the genetically determined gut microbiota on the mediator (β1) via univariable Mendelian randomization (UVMR). Subsequently, the causal effect of the mediator on OA and fractures was estimated. Thereafter, the total effect of gut microbiota on OA and fractures, mediated by each mediator, was calculated by dividing the indirect effect, which was obtained by multiplying the outcomes from the two steps (β1 × β2 pooled) by the total effect. The delta method was used to derive standard errors from the two-sample MR analyses.

Further, a colocalization analysis was performed to confirm whether the significantly mediated microbiota shared a causal variant common to both neurophysiological states and OA and fractures. The posterior probability of a specific variant (±10,000 bp) was colocalized with summary statistics of neurophysiological states, OA, and fractures [42].

All MR analyses were performed using the two-sample MR package in R, version 4.3.0 (R Foundation for Statistic Computing, Vienna, Austria). The LDSC was conducted using the ldscr package in R version 0.1.0. The colocalization test was performed using the coloc package in R version 5.2.2.

## 3. Results

### 3.1. MR Results

**Figure 2:**
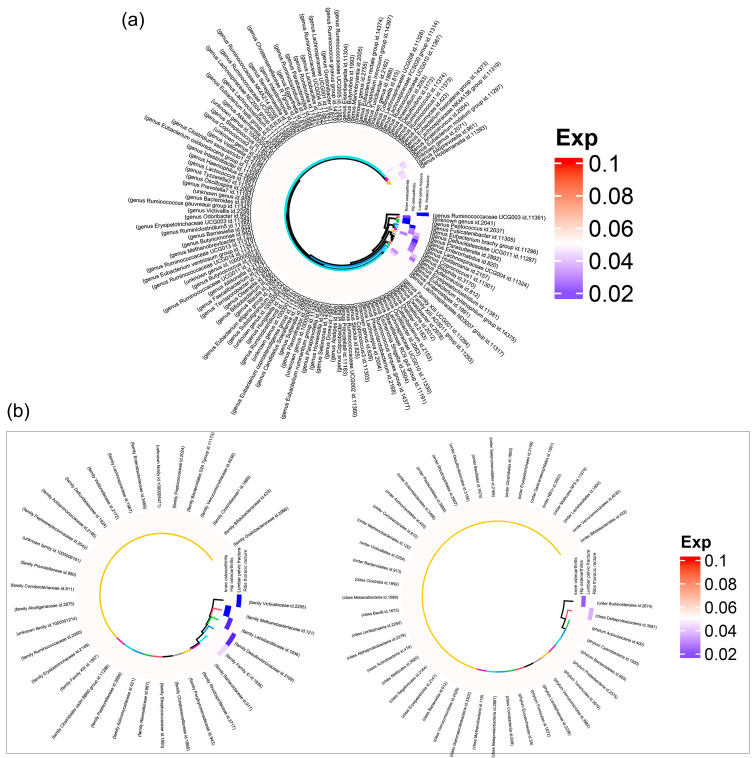
Heatmap displaying causalities of 35 families and 131 genera. (**a**) Causal relationship between 131 gut microbiota genera and bone diseases. (**b**) Causative roles of 9 phyla, 16 classes, 20 orders, and 35 families within the gut microbiota in bone diseases.

MR analysis provided significant insights into the relationship between gut microbiota and orthopedic conditions. Ten and six taxa were causally associated with hip and knee OA, respectively. Notably, one taxon was found to be associated with both hip and knee OA. Examinations of the lumbar pelvic and rib–sternum–thoracic vertebral fractures were influenced by eight and nine taxa, respectively. Notably, the taxa influencing these fracture types did not overlap (Figure 2).

IVW estimates were used to determine the causal effects of the microbiota on hip and knee OA, which were effective in the absence of heterogeneity or pleiotropy. The Ruminococcaceae genus was associated with negative effects on both hip (OR: 0.789, 95% CI: [0.700, 0.891], *p* = 0.001) and knee (OR: 0.880, 95% CI: [0.800, 0.969], *p* = 0.009) OA. In contrast, the Victivallaceae family showed a unique positive causal association with hip OA (OR: 1.091, 95% CI: [1.028, 1.157], *p* = 0.004). 

The Deltaproteobacteria class (OR: 0.892, 95% CI: [0.801,0.993], *p* = 0.036), family XI (OR: 0.945, 95% CI: [0.894, 0.998], *p* = 0.043), and the Desulfovibrionaceae family (OR: 0.859, 95% CI: [0.760, 0.970], *p* = 0.014) negatively influenced knee OA.

**Figure 3:**
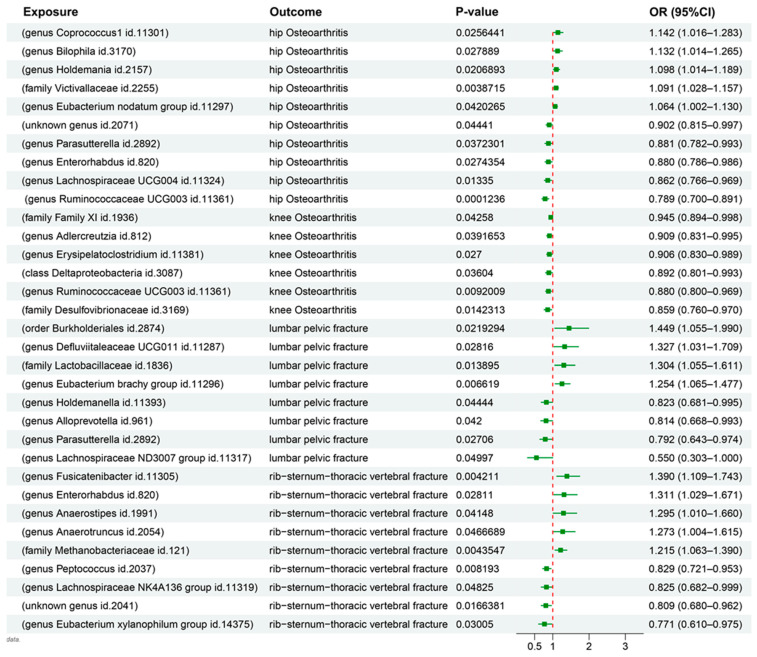
Forest plot representing the results of MR analysis. Individual estimates of the causal relationship between gut microbiota and bone diseases, derived from MR. Each result is represented by boxes and corresponding confidence bars. Only gut microbiota with an inverse variance weight (IVW) < 0.05 were included in the analysis.

The Lactobacillaceae family exerted a strong positive causal influence on lumbar pelvic fractures (OR: 1.304, 95% CI: [1.055, 1.611], *p* = 0.014). Similarly, the Methanobacteriaceae family demonstrated a positive causal effect on rib–sternum–thoracic vertebral fractures (OR: 1.215, 95% CI: [1.063, 1.390], *p* = 0.004). Further details regarding other relevant taxa are presented in Figure 3.

### 3.2. LDSC Results

**Figure 4:**
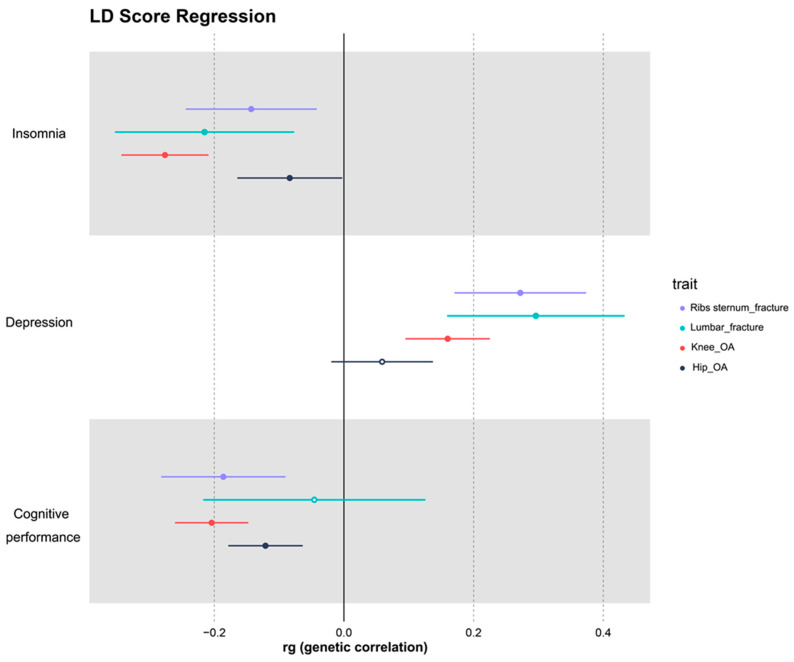
A forest plot displaying LDSC results. Results of the LDSC analysis that explored the correlations between neurophysiological states and bone diseases, with the x-axis signifying the genetic correlation (rg). The dots and their corresponding horizontal lines represent specific rg values and their respective 95% confidence intervals. Hollow dots indicate *p* > 0.05, whereas solid dots denote *p* < 0.05.

The results of LDSC analysis confirmed the genetic correlation (rg) between various neurophysiological states and orthopedic conditions. Insomnia was significantly correlated with knee (rg: 0.276, *p* = 0.001) and hip (rg: 0.084, *p* = 0.043) OA and rib–sternum–thoracic vertebral (rg: 0.143, *p* = 0.005) and lumbar pelvic (rg: 0.215, *p* = 0.002) fractures. Similarly, depression was genetically linked to knee OA (rg: 0.160, *p* = 0.001) and rib–sternum–thoracic vertebral (rg: 0.272, *p* = 0.001) and lumbar pelvic (rg: 0.296, *p* = 0.001) fractures. However, no significant genetic correlation was detected between depression and hip OA (rg: 0.0594, *p* = 0.141). Cognitive performance was significantly correlated with knee (rg: 0.204, *p* = 0.001) and hip (rg: 0.121, *p* = 0.001) OA and rib–sternum–thoracic vertebral fractures (rg: 0.186, *p* = 0.001). Notably, no significant correlation was observed between cognitive performance and lumbar pelvic fractures (rg: 0.046, *p* = 0.601) (Figure 4).

### 3.3. Mediation MR Results

The mediation analysis began with an estimation of the causal effect of genetically determined gut microbiota on the mediator, using UVMR, and was denoted as β1. In this analysis, the gut microbiota was considered the exposure factor, whereas cognitive performance, depression, and insomnia were evaluated as outcome variables in the calculation of this causal effect.

In scenarios where heterogeneity or pleiotropy was absent, the IVW estimates were favored. The findings did not indicate a causal influence of cognitive performance on hip OA (β: −0.153, 95% CI: [−0.327, 0.021], *p* = 0.084). Owing to the presence of heterogeneity in knee OA, the weighted median method was employed, which revealed a causal effect (β: −0.267, 95% CI: [−0.404, −0.128], *p* = 1.5 × 10^−5^). Using IVW estimates, we determined that cognitive performance exerted causal effects on lumbar pelvic (β: −0.319, 95% CI: [−0.593, −0.045], *p* = 0.023) and rib–sternum–thoracic vertebral (β: −0.256, 95% CI: [−0.487, −0.026], *p* = 0.029) fractures.

To examine the role of insomnia as a mediator of bone disease, IVW estimation was used in the absence of heterogeneity to ascertain its effects. The data revealed that insomnia exerted a causal impact on hip (β: 0.568, 95% CI: [0.173, 0.963], *p* = 0.005) and knee (β: 0.835, 95% CI: [0.373, 1.297], *p* = 0.001) OA; however, the effects on rib–sternum–thoracic vertebral (β: 0.643, 95% CI: [−0.107, 1.393], *p* = 0.092) and lumbar pelvic (β: 0.215, 95% CI: [−0.676, 1.106], *p* = 0.637) fractures were not statistically significant.

No causal relationship was detected between depression, fractures, and hip OA. However, a significant causal relationship was found exclusively between depression and knee OA (β: 1.146, 95% CI: [0.135, 2.157], *p* = 0.026).

**Figure 5:**
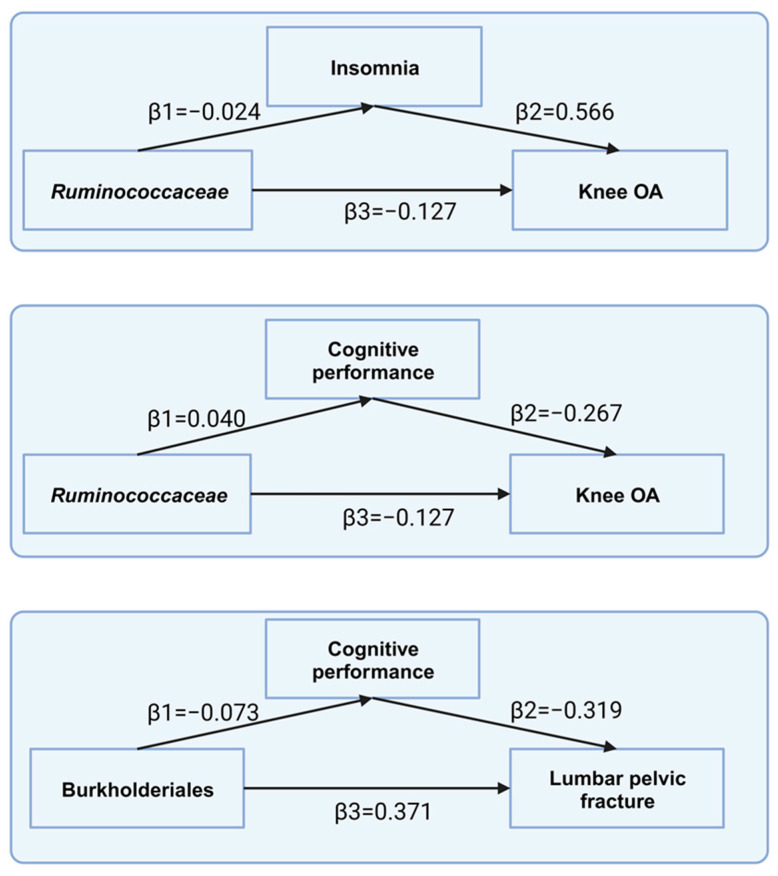
Mediating effects of gut microbiota on bone diseases. β1 signifies the causal influence of Ruminococcaceae or Burkholderiales on potential mediators, particularly insomnia or cognitive performance. β2 depicts the causal effect of these mediators, either insomnia or cognitive performance, on bone diseases. β3 represents the cumulative causal impact of gut microbiota on bone disease.

To conduct an effective mediation analysis, the taxa under investigation should exert a significant influence on both the mediator and outcome variables. In accordance with this principle, Ruminococcaceae demonstrated a comprehensive causal effect on knee OA and a significant impact on the mediator (β: −0.040, 95% CI: [−0.011, −0.068], *p* = 0.007). This result suggests that cognitive performance mediates 8.41% of the causal effects of Ruminococcaceae on knee OA. In addition, insomnia mediated 10.7% of the causal effects of Ruminococcaceae on knee OA. Similarly, the order Burkholderiales demonstrated a total causal effect on lumbar pelvic fractures and significant causal effects on cognitive performance (β: −0.073, 95% CI: [−0.124, −0.023], *p* = 0.004). This result implies that cognitive performance mediated 6.28% of the causal effect of Burkholderiales on lumbar fractures. However, no evidence suggested a mediating role of depression in the relationship between the gut microbiota and bone diseases (Figure 5).

### 3.4. Gene Colocalization Results

**Figure 6:**
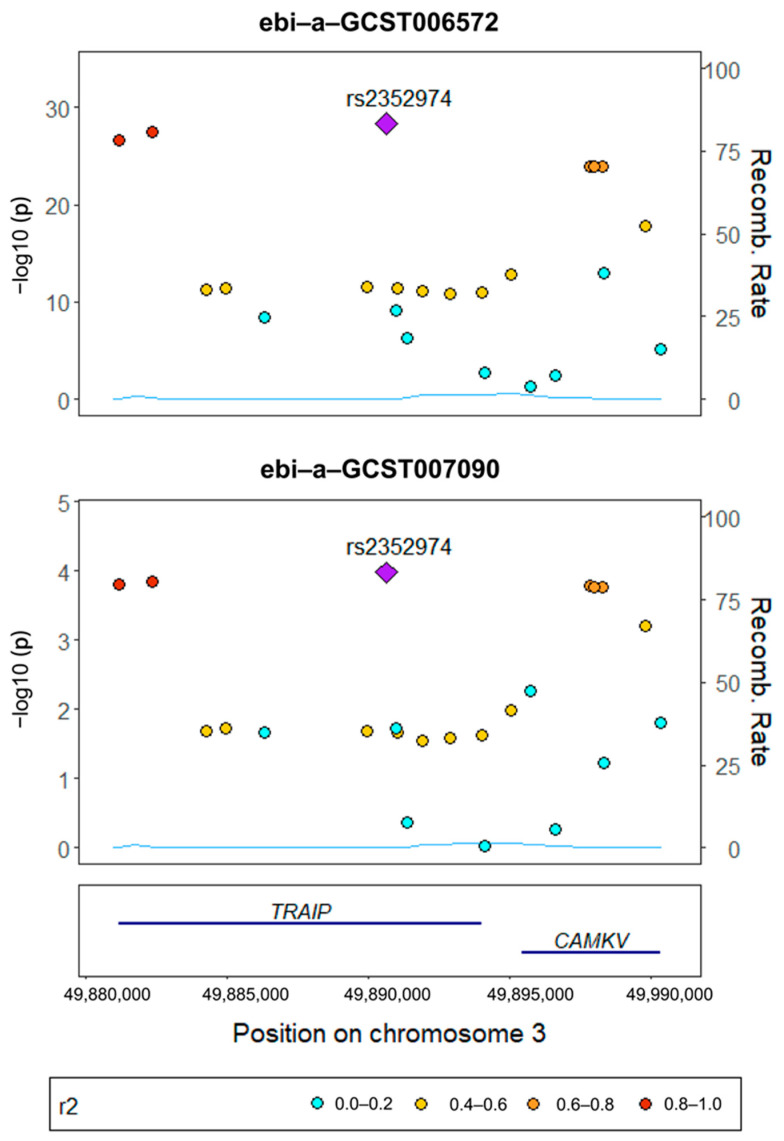
Results of gene colocalization analysis. Identifiers ebi-a-GCST006572 and ebi-a-GCS007090, sourced from the Integrative Epidemiology Unit OpenGWAS project, correspond to cognitive performance and knee osteoarthritis (OA), respectively. Notably, rs2352974, a shared variant, was present under both conditions. This variant is located within the TRAF-interacting protein (TRAIP) locus on chromosome 3.

The evidence for colocalization was closely examined within the 10,000 bp region surrounding SNP rs2352974 at the TRAF-interacting protein (TRAIP) locus. This analysis used summary statistics for cognitive performance and knee OA. The colocalization posterior probability (PP_H4) due to COLOC was high for both cognitive performance and knee OA (PP_H4 > 0.9), suggesting that these two conditions share a common variant, rs2352974. However, no shared genetic loci were identified for other gut microbiota, neurophysiological states, or bone-related diseases (Figure 6).

## 4. Discussion

To the best of our knowledge, this is the first large-scale MR analysis to establish a comprehensive causal relationship between the gut microbiota, neurophysiological states, and bone health. Although previous observational studies suggested this association, they did not conclusively establish causality, owing to potential confounders and reverse causality [15]. This study identified specific microbiota alterations with the potential to enhance cognitive performance and reduce insomnia, fracture risk, and OA. This nuanced understanding underscores the human body as a system of interconnected yet distinct parts.

The composition of the gut microbiota, which fluctuates across the lifespan of an individual or among different individuals, is influenced by numerous factors. The resultant dynamic modifications in microbiota composition can affect health or disease susceptibility by altering its diversity or composition. Consequently, elucidating the intricate and comprehensive mechanisms driving the dialog between the disease and its associated gut microbiota is important. The communication mechanism between the gut, its resident flora, and various organs, known as the gut–organ axis, has been increasingly recognized as critical for maintaining the health of different organs [43]. Notably, a quintessential example of this relationship is evident in periodontitis. As one of the most common oral diseases leading to tooth loss among adults, periodontitis results in the degradation of the tissues supporting teeth. Untreated, this can lead to tooth looseness and eventual loss [44]. The mouth and gut serve as the initial and terminal sites of the digestive tract microbiome and share some common microbial inhabitants. This ‘oral–gut axis’ suggests that a balanced gut microbiome could be beneficial in controlling periodontitis [45]. Furthermore, the administration of gut probiotics having anti-inflammatory properties is emerging as a new approach to address bacterial imbalances in periodontitis and to prevent bone loss [46]. This interplay elucidates a multifaceted relationship between localized and systemic conditions, emphasizing the need to consider gut health in the comprehensive management of diseases with broader systemic implications [47].

Recent studies have revealed a possible biological interplay between the gut, brain, and bones. Osteocalcin (OCN) is a protein secreted by osteoblasts that regulates brain function. It can penetrate the blood–brain barrier and directly bind to neurons in the hippocampal CA3 region, which restored cognitive function in mice [48]. However, when the gut microbiota was eliminated via antibiotics before OCN administration, the protective effects of the protein against dopaminergic neuron damage and improvements in motor function in mice were no longer effective. These observations suggest that the neuroprotective effect of OCN in mice is mediated by the gut microbiota [49]. Such findings further strengthen our understanding of the gut–brain–bone axis [50].

Ruminococcaceae is a dominant bacterial genus within the human gut, with the primary function of breaking down complex dietary and host-derived carbohydrates into short-chain fatty acids (SCFAs) [51], such as butyric acid. SCFAs are a vital energy source for cells within the intestine and confer wide-ranging health benefits, including anti-inflammatory effects and the enhancement of intestinal barrier function. These findings suggest that Ruminococcaceae bacteria have the potential to enhance cognitive function, mitigate insomnia, and prevent OA. These positive outcomes can be attributed to the regulatory influence of SCFAs, particularly butyrate, on inflammatory responses and their potential beneficial effects on brain health. Conversely, based on these findings, Burkholderiales, despite their low abundance in the human gut, may have a significant impact on reducing fracture risk and enhancing cognitive performance [52]. Burkholderiales possess the unique ability to degrade a wide array of compounds, including xenobiotics, which can assist in eliminating potentially osteotoxic substances. Simultaneously, certain *Burkholderia* species that act as opportunistic pathogens may increase the diversity of the gut microbiota, thereby indirectly supporting bone health [53]. However, considering the limited current understanding of these bacteria, their intricate interactions, and their exact roles in human health, a more comprehensive investigation is necessary.

This study had some limitations. Although the MR analysis uncovered several significant associations between gut microbiota taxa and both OA and fracture risk, these findings were not mirrored by the LDSC analysis. The LDSC results did not reveal a substantial genetic correlation between the gut microbiota and bone diseases, primarily because of the low heritability of the gut microbiota (H2_P > 0.05). The composition of the gut microbiota is greatly influenced by environmental factors such as diet, lifestyle, and medication [54]. These variables can considerably affect the composition and function of the gut microbiota, thereby overshadowing potential genetic influences. Therefore, the absence of a substantial genetic correlation does not negate the potential connection between the gut microbiota and bone diseases. Notably, it highlights the intricate interplay between genetic and environmental factors in shaping the gut microbiota [55]. A more comprehensive approach to future research could yield valuable insights. For instance, microbiome-wide association studies could account for environmental influences, providing a more holistic understanding of the relationship between gut microbiota and bone health. Furthermore, investigating potential mechanisms through which the gut microbiota might influence bone diseases, such as the modulation of the immune response or the production of metabolites affecting bone metabolism, could offer significant insights.

Gene colocalization analysis revealed an unexpected finding: a specific locus within the TRAIP gene that exhibited a significant correlation with two seemingly unrelated conditions, cognitive performance and knee OA. Considering the critical role of TRAIP in cell cycle regulation and DNA repair processes, it is feasible that TRAIP function directly influences the progression of OA and the associated DNA anomalies [56]. However, the precise role and mechanistic contribution of TRAIP under these conditions remain largely elusive and present an intriguing avenue for future rigorous functional studies. Employing relevant cellular or animal models could elucidate these uncertainties and provide more insight into the complex roles of TRAIP under these conditions. Moreover, the unique properties of TRAIP offer intriguing theoretical support for its potential as a therapeutic target, paving the way for exciting new fields of scientific investigation [57]. In the future, this domain is expected to yield groundbreaking discoveries.

The MR method offers a near-random context for observing the effects of the gut microbiota, neurophysiological states, and bone diseases. This approach lessens the impact of confounding factors, operating on the assumptions that IVs connect with the outcome exclusively via the exposure variable, IVs are unrelated to confounding factors, and no hidden direct impact exists between the IV and outcome [58]. If these assumptions are violated, it could result in bias when estimating causal relationships. Therefore, although robust evidence was provided, the findings should be validated using alternative research methods. Another limitation of this study was the exclusive inclusion of European participants. Therefore, it remains uncertain whether the observed causal relationships between gut microbiota, neurophysiological states, and bone diseases are applicable to other demographic populations. Finally, the mediating variables in this investigation accounted for only 5–11% of the relationships between the gut microbiota and fractures or OA. Despite being statistically significant, the effect was modest, underscoring that the mediating variable plays a limited role in elucidating the link between the gut microbiota and these conditions. Consequently, other unidentified pathways may exist through which the gut microbiota affect bone diseases.

Despite these limitations, the findings of this study shed new light on the role of the gut–bone–brain axis in maintaining skeletal health, a critical factor that could significantly shape healthcare strategies in older populations. The discovery of causal relationships between the gut microbiota and cognitive performance, insomnia, OA, and fractures has potential implications for public health policy, including the implementation of preventative measures and timely intervention strategies. Enhancing the gut microbiota not only improves cognitive performance and alleviates insomnia, but also decreases the incidence of hip and knee OA and site-specific fractures. These insights provide a crucial foundation for the development of future clinical interventions.

## 5. Conclusions

The findings of this study provide compelling evidence that modifications to the gut microbiota can enhance cognitive abilities and mitigate the symptoms of insomnia, potentially reducing the incidence of site-specific fractures and OA. These results suggest that the development of strategies to optimize the gut microbiota could represent innovative and effective methods for fracture and OA prevention. However, additional research is crucial to decipher the complex biological interactions between the gut microbiota, neurophysiological conditions, and bone health. Such an exploration will facilitate the comprehensive use of the potential benefits nested within this relationship. 

## Data Availability

The data described in this article have been sourced from two online databases, namely the MiBioGen consortium and the IEU OpenGWAS project. MiBioGen consortium is accessible at the GCC website [https://mibiogen.gcc.rug.nl/menu/main/home/ (accessed on 1 July 2023)]. The IEU OpenGWAS project is accessible at the GWAS Catalog website [https://gwas.mrcieu.ac.uk/ (accessed on 15 July 2023)].

## References

[1] Guo J., Huang X., Dou L., Yan M., Shen T., Tang W., Li J. (2022). Aging and aging-related diseases: From molecular mechanisms to interventions and treatments. Signal Transduct. Target. Ther..

[2] Vestergaard P., Rejnmark L., Mosekilde L. (2009). Osteoarthritis and Risk of Fractures. Calcif. Tissue Int..

[3] Tong L., Yu H., Huang X., Shen J., Xiao G., Chen L., Wang H., Xing L., Chen D. (2022). Current understanding of osteoarthritis pathogenesis and relevant new approaches. Bone Res..

[4] Martel-Pelletier J., Barr A.J., Cicuttini F.M., Conaghan P.G., Cooper C., Goldring M.B., Goldring S.R., Jones G., Teichtahl A.J., Pelletier J.-P. (2016). Osteoarthritis. Nat. Rev. Dis. Primers.

[5] Bhanushali A., Kovoor J.G., Stretton B., Kieu J.T., Bright R.A., Hewitt J.N., Ovenden C.D., Gupta A.K., Afzal M.Z., Edwards S. (2022). Outcomes of early versus delayed weight-bearing with intramedullary nailing of tibial shaft fractures: A systematic review and meta-analysis. Eur. J. Trauma. Emerg. Surg..

[6] Rubenstein L.Z. (2006). Falls in older people: Epidemiology, risk factors and strategies for prevention. Age Ageing.

[7] Fang E.F., Xie C., Schenkel J.A., Wu C., Long Q., Cui H., Aman Y., Frank J., Liao J., Zou H. (2020). A research agenda for ageing in China in the 21st century (2nd edition): Focusing on basic and translational research, long-term care, policy and social networks. Ageing Res. Rev..

[8] Fairley J.L., Seneviwickrama M., Yeh S., Anthony S., Chou L., Cicuttini F.M., Sullivan K., Briggs A.M., Wluka A.E. (2021). Person-centred care in osteoarthritis and inflammatory arthritis: A scoping review of people’s needs outside of healthcare. BMC Musculoskelet. Disord..

[9] Vincent H.K., Horodyski M., Vincent K.R., Brisbane S.T., Sadasivan K.K. (2015). Psychological Distress After Orthopedic Trauma: Prevalence in Patients and Implications for Rehabilitation. PM&R.

[10] Sekirov I., Russell S.L., Antunes L.C., Finlay B.B. (2010). Gut microbiota in health and disease. Physiol. Rev..

[11] Hou K., Wu Z.-X., Chen X.-Y., Wang J.-Q., Zhang D., Xiao C., Zhu D., Koya J.B., Wei L., Li J. (2022). Microbiota in health and diseases. Signal Transduct. Target. Ther..

[12] Yang W., Cong Y. (2021). Gut microbiota-derived metabolites in the regulation of host immune responses and immune-related inflammatory diseases. Cell. Mol. Immunol..

[13] Peng J., Yu X.-J., Yu L.-L., Tian F.-W., Zhao J.-X., Zhang H., Chen W., Zhai Q.-X. (2021). The influence of gut microbiome on bone health and related dietary strategies against bone dysfunctions. Food Res. Int..

[14] Chen Y.C., Greenbaum J., Shen H., Deng H.W. (2017). Association Between Gut Microbiota and Bone Health: Potential Mechanisms and Prospective. J. Clin. Endocrinol. Metab..

[15] Lu L., Chen X., Liu Y., Yu X. (2021). Gut microbiota and bone metabolism. FASEB J..

[16] Blaak E.E., Canfora E.E., Theis S., Frost G., Groen A.K., Mithieux G., Nauta A., Scott K., Stahl B., van Harsselaar J. (2020). Short chain fatty acids in human gut and metabolic health. Benef. Microbes.

[17] Medawar E., Haange S.-B., Rolle-Kampczyk U., Engelmann B., Dietrich A., Thieleking R., Wiegank C., Fries C., Horstmann A., Villringer A. (2021). Gut microbiota link dietary fiber intake and short-chain fatty acid metabolism with eating behavior. Transl. Psychiatry.

[18] Seely K.D., Kotelko C.A., Douglas H., Bealer B., Brooks A.E. (2021). The Human Gut Microbiota: A Key Mediator of Osteoporosis and Osteogenesis. Int. J. Mol. Sci..

[19] Kesika P., Suganthy N., Sivamaruthi B.S., Chaiyasut C. (2021). Role of gut-brain axis, gut microbial composition, and probiotic intervention in Alzheimer’s disease. Life Sci..

[20] Morais L.H., Schreiber H.L., Mazmanian S.K. (2021). The gut microbiota–brain axis in behaviour and brain disorders. Nat. Rev. Microbiol..

[21] Ma Q., Xing C., Long W., Wang H.Y., Liu Q., Wang R.-F. (2019). Impact of microbiota on central nervous system and neurological diseases: The gut-brain axis. J. Neuroinflammation.

[22] Roos P.M. (2014). Osteoporosis in neurodegeneration. J. Trace Elem. Med. Biol..

[23] He Y., Li Z., Alexander P.G., Ocasio-Nieves B.D., Yocum L., Lin H., Tuan R.S. (2020). Pathogenesis of Osteoarthritis: Risk Factors, Regulatory Pathways in Chondrocytes, and Experimental Models. Biology.

[24] Ni Z., Zhou S., Li S., Kuang L., Chen H., Luo X., Ouyang J., He M., Du X., Chen L. (2020). Exosomes: Roles and therapeutic potential in osteoarthritis. Bone Res..

[25] Ramsey N.F., Jansma J.M., Jager G., Van Raalten T., Kahn R.S. (2004). Neurophysiological factors in human information processing capacity. Brain.

[26] Xie N., Wang Z., Shu Q., Liang X., Wang J., Wu K., Nie Y., Shi Y., Fan D., Wu J. (2023). Association between Gut Microbiota and Digestive System Cancers: A Bidirectional Two-Sample Mendelian Randomization Study. Nutrients.

[27] Wang F., Li N., Ni S., Min Y., Wei K., Sun H., Fu Y., Liu Y., Lv D. (2023). The Effects of Specific Gut Microbiota and Metabolites on IgA Nephropathy-Based on Mendelian Randomization and Clinical Validation. Nutrients.

[28] Sanchez-Lopez E., Coras R., Torres A., Lane N.E., Guma M. (2022). Synovial inflammation in osteoarthritis progression. Nat. Rev. Rheumatol..

[29] Glyn-Jones S., Palmer A.J., Agricola R., Price A.J., Vincent T.L., Weinans H., Carr A.J. (2015). Osteoarthritis. Lancet.

[30] Mahmoudian A., Lohmander L.S., Mobasheri A., Englund M., Luyten F.P. (2021). Early-stage symptomatic osteoarthritis of the knee—Time for action. Nat. Rev. Rheumatol..

[31] Barrio C., Arias-Sánchez S., Martín-Monzón I. (2022). The gut microbiota-brain axis, psychobiotics and its influence on brain and behaviour: A systematic review. Psychoneuroendocrinology.

[32] Pettit R.W., Amos C.I. (2022). Linkage Disequilibrium Score Statistic Regression for Identifying Novel Trait Associations. Curr. Epidemiol. Rep..

[33] Zeng P., Shao Z., Zhou X. (2021). Statistical methods for mediation analysis in the era of high-throughput genomics: Current successes and future challenges. Comput. Struct. Biotechnol. J..

[34] Claus S.P., Ellero S.L., Berger B., Krause L., Bruttin A., Molina J., Paris A., Want E.J., de Waziers I., Cloarec O. (2011). Colonization-induced host-gut microbial metabolic interaction. mBio.

[35] Skrivankova V.W., Richmond R.C., Woolf B.A.R., Yarmolinsky J., Davies N.M., Swanson S.A., VanderWeele T.J., Higgins J.P.T., Timpson N.J., Dimou N. (2021). Strengthening the Reporting of Observational Studies in Epidemiology Using Mendelian Randomization: The STROBE-MR Statement. JAMA.

[36] Liu X., Tang S., Zhong H., Tong X., Jie Z., Ding Q., Wang D., Guo R., Xiao L., Xu X. (2021). A genome-wide association study for gut metagenome in Chinese adults illuminates complex diseases. Cell Discov..

[37] Lee J.J., Wedow R., Okbay A., Kong E., Maghzian O., Zacher M., Nguyen-Viet T.A., Bowers P., Sidorenko J., Karlsson Linnér R. (2018). Gene discovery and polygenic prediction from a genome-wide association study of educational attainment in 1.1 million individuals. Nat. Genet..

[38] Howard D.M., Adams M.J., Shirali M., Clarke T.K., Marioni R.E., Davies G., Coleman J.R.I., Alloza C., Shen X., Barbu M.C. (2018). Genome-wide association study of depression phenotypes in UK Biobank identifies variants in excitatory synaptic pathways. Nat. Commun..

[39] Song W., Torous J., Kossowsky J., Chen C.-Y., Huang H., Wright A. (2020). Genome-wide association analysis of insomnia using data from Partners Biobank. Sci. Rep..

[40] Burgess S., Davey Smith G., Davies N.M., Dudbridge F., Gill D., Glymour M.M., Hartwig F.P., Kutalik Z., Holmes M.V., Minelli C. (2019). Guidelines for performing Mendelian randomization investigations: Update for summer 2023. Wellcome Open Res..

[41] Hou T., Dai H., Wang Q., Hou Y., Zhang X., Lin H., Wang S., Li M., Zhao Z., Lu J. (2023). Dissecting the causal effect between gut microbiota, DHA, and urate metabolism: A large-scale bidirectional Mendelian randomization. Front. Immunol..

[42] Foley C.N., Staley J.R., Breen P.G., Sun B.B., Kirk P.D.W., Burgess S., Howson J.M.M. (2021). A fast and efficient colocalization algorithm for identifying shared genetic risk factors across multiple traits. Nat. Commun..

[43] Guo Y., Chen X., Gong P., Li G., Yao W., Yang W. (2023). The Gut-Organ-Axis Concept: Advances the Application of Gut-on-Chip Technology. Int. J. Mol. Sci..

[44] Petersen P.E., Ogawa H. (2012). The global burden of periodontal disease: Towards integration with chronic disease prevention and control. Periodontology 2000.

[45] Hajishengallis G., Lamont R.J. (2012). Beyond the red complex and into more complexity: The polymicrobial synergy and dysbiosis (PSD) model of periodontal disease etiology. Mol. Oral Microbiol..

[46] Ikram S., Hassan N., Raffat M.A., Mirza S., Akram Z. (2018). Systematic review and meta-analysis of double-blind, placebo-controlled, randomized clinical trials using probiotics in chronic periodontitis. J. Investig. Clin. Dent..

[47] Zhou T., Xu W., Wang Q., Jiang C., Li H., Chao Y., Sun Y. (2023). The effect of the “Oral-Gut” axis on periodontitis in inflammatory bowel disease: A review of microbe and immune mechanism associations. Front. Cell. Infect. Microbiol..

[48] Han Y., You X., Xing W., Zhang Z., Zou W. (2018). Paracrine and endocrine actions of bone—The functions of secretory proteins from osteoblasts, osteocytes, and osteoclasts. Bone Res..

[49] Hou Y.F., Shan C., Zhuang S.Y., Zhuang Q.Q., Ghosh A., Zhu K.C., Kong X.K., Wang S.M., Gong Y.L., Yang Y.Y. (2021). Gut microbiota-derived propionate mediates the neuroprotective effect of osteocalcin in a mouse model of Parkinson’s disease. Microbiome.

[50] Zhou R., Guo Q., Xiao Y., Guo Q., Huang Y., Li C., Luo X. (2021). Endocrine role of bone in the regulation of energy metabolism. Bone Res..

[51] La Reau A.J., Suen G. (2018). The Ruminococci: Key symbionts of the gut ecosystem. J. Microbiol..

[52] Thursby E., Juge N. (2017). Introduction to the human gut microbiota. Biochem. J..

[53] Rojas-Rojas F.U., López-Sánchez D., Meza-Radilla G., Méndez-Canarios A., Ibarra J.A., Estrada-de Los Santos P. (2019). The controversial Burkholderia cepacia complex, a group of plant growth promoting species and plant, animals and human pathogens. Rev. Argent. Microbiol..

[54] Hasan N., Yang H. (2019). Factors affecting the composition of the gut microbiota, and its modulation. PeerJ.

[55] Grieneisen L., Dasari M., Gould T.J., Björk J.R., Grenier J.-C., Yotova V., Jansen D., Gottel N., Gordon J.B., Learn N.H. (2021). Gut microbiome heritability is nearly universal but environmentally contingent. Science.

[56] Harley M.E., Murina O., Leitch A., Higgs M.R., Bicknell L.S., Yigit G., Blackford A.N., Zlatanou A., Mackenzie K.J., Reddy K. (2016). TRAIP promotes DNA damage response during genome replication and is mutated in primordial dwarfism. Nat. Genet..

[57] Feng W., Guo Y., Huang J., Deng Y., Zang J., Huen M.S.-Y. (2016). TRAIP regulates replication fork recovery and progression via PCNA. Cell Discov..

[58] Sanderson E., Glymour M.M., Holmes M.V., Kang H., Morrison J., Munafò M.R., Palmer T., Schooling C.M., Wallace C., Zhao Q. (2022). Mendelian randomization. Nat. Rev. Methods Primers.

